# Local Josephson vortex generation and manipulation with a Magnetic Force Microscope

**DOI:** 10.1038/s41467-019-11924-0

**Published:** 2019-09-05

**Authors:** Viacheslav V. Dremov, Sergey Yu. Grebenchuk, Andrey G. Shishkin, Denis S. Baranov, Razmik A. Hovhannisyan, Olga V. Skryabina, Nickolay Lebedev, Igor A. Golovchanskiy, Vladimir I. Chichkov, Christophe Brun, Tristan Cren, Vladimir M. Krasnov, Alexander A. Golubov, Dimitri Roditchev, Vasily S. Stolyarov

**Affiliations:** 1 Moscow Institute of Physics and Technology, 141700 Dolgoprudny, Russia; 2 Dukhov Research Institute of Automatics (VNIIA), 127055 Moscow, Russia; 3 Institute of Solid State Physics RAS, 142432 Chernogolovka, Russia; 4 LPEM, ESPCI Paris, PSL Research University, CNRS, 75005 Paris, France; 5 National University of Science and Technology MISIS, 119049 Moscow, Russia; 6 Institut des Nanosciences de Paris, INSP, UMR-7588, Sorbonne University, CNRS, 75005 Paris, France; 7 Department of Physics, Stockholm University, AlbaNova University Center, SE-10691 Stockholm, Sweden; 8 Faculty of Science and Technology and MESA+ Institute of Nanotechnology, 7500AE Enschede, The Netherlands; 9 Sorbonne Universite, CNRS, LPEM, 75005 Paris, France; 10 Donostia International Physics Center (DIPC), 20018 San Sebastin/Donostia, Basque, Spain; 11 Solid State Physics Department, Kazan Federal University, 420008 Kazan, Russia

**Keywords:** Superconducting properties and materials, Superconducting devices, Atomic force microscopy

## Abstract

Josephson vortices play an essential role in superconducting quantum electronics devices. Often seen as purely conceptual topological objects, 2*π*-phase singularities, their observation and manipulation are challenging. Here we show that in Superconductor—Normal metal—Superconductor lateral junctions Josephson vortices have a peculiar magnetic fingerprint that we reveal in Magnetic Force Microscopy (MFM) experiments. Based on this discovery, we demonstrate the possibility of the Josephson vortex generation and manipulation by the magnetic tip of a MFM, thus paving a way for the remote inspection and control of individual nano-components of superconducting quantum circuits.

## Abstract (web summary)

Josephson vortices (JVs) play an important role in superconducting quantum devices, but they remain difficult to be observed and manipulated. Here, Dremov et al. report magnetic fingerprint of JVs in magnetic force microscopy experiments, which paves a way to generate and control JVs.

## Introduction

The variety of available ultra-sensitive superconducting devices, qubits, and architectures for quantum computing is rapidly growing. Superconducting quantum electronics (SQE)^1,2^ devices are expected to challenge the conventional semiconducting ones in the near future^3^. The Josephson junctions (JJs) are building blocks of the SQE; they are composed of two superconducting leads linked by a short non-superconducting barrier. The properties of JJs are sensitive to the junction geometry, used materials, temperature, applied supercurrents, magnetic fields, etc. These parameters determine the quantum phase portrait of the superconducting correlations inside and in the vicinity of the JJ.

Due to the spatial coherence of the superconducting condensate, the quantum phase portraits of conventional s-wave superconductors may only contain 2*π*-phase loops or multiple. Single 2*π*-singularities located in the superconducting electrodes are associated with the Abrikosov vortices, those located inside the links with the Josephson ones^4^. The integer number *n* of Josephson vortices present in a JJ is associated with the *n*-th branch of Fraunhofer-type modulation of the critical current vs magnetic field *I*_c_(*H*).

Unlike Abrikosov vortices, which were revealed by Scanning Tunneling Microscopy and Spectroscopy (STM/STS) already in 1989 owing their normal cores^5^, the investigation of core-less Josephson ones by STM/STS is more difficult^6–9^. Scanning SQUID experiments were more successful in revealing a strong screening length anisotropy of interlayer vortices in high-*T*_C_ superconductors^10^ or in studying vortices pinned at grain boundaries^11–14^. These seminal works provided first strong evidences for a *d*-wave pairing in cuprates. Though, because of strong pinning and short spatial scales in high-*T*_C_ materials, these works did not address a more general problem of local generation, dynamics and manipulation of Josephson vortices inside JJs.

Lateral (planar) JJs are very promising for both basic research and applications^15–18^ even if they are not as widely used as traditional sandwich-like (overlap) multilayer JJs^19–22^. The planar geometry enables a great flexibility in designing new types of devices with a large number of foreseen applications, including single-photon detection^23^, measurement of magnetic flux induced by atomic spins^24^, nano-electronic measurements^25^. Planar JJs can be made by different techniques and with various barrier materials, including normal metals, ferromagnets, two-dimensional electron gas, graphene, and topological insulators^26–29^. Importantly, the lateral geometry of JJs makes them suitable for studies by scanning probe microscopies and spectroscopies, such as STM/STS^8,30–32^, Scanning SQUID^33,34^ or Magnetic Force Microscopy (MFM)^35^, as we do in this work.

The MFM is a convenient tool for probing superconducting properties in the real space and with nanometer resolution, such as the London penetration depth^36,37^, Abrikosov vortices^35,38,39^, and domain structures in ferromagnetic superconductors^40–42^. Recent development of MFM-based methods enabled the study of superconducting phase slips^43–45^.

**Fig. 1:**
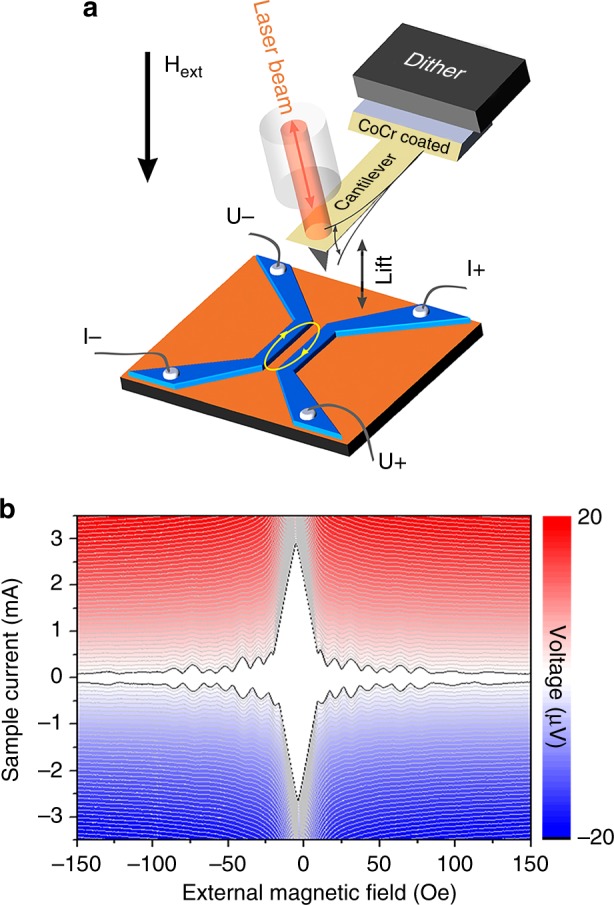
Design and electronic characteristics of the studied SNS device. **a** experimental setup: 100-nm-thick Nb leads (in blue) are patterned on a 50-nm-thick Cu layer (in orange); the leads are bonded for transport measurements. The ellipse marks the junction region 2500 nm × 200 nm. The MFM cantilever with a Co/Cr-coated tip oscillates, excited by a dither; an optical fiber is used for the oscillation readout; **b** “Fraunhofer pattern” of the device: the voltage drop across the junction is measured as a function of applied current and external magnetic field (the MFM tip is retracted far away from the device). Red (blue): positive (negative) voltage drop; white: zero-voltage drop representing the superconducting state

In the present work we apply MFM (see Methods: AFM and MFM experiment) to reveal static and dynamic responses of Josephson vortices in planar Nb/Cu/Nb JJs^9^. Figure 1a sketches the device and the scheme of the experiment. The device fabrication is described in Methods: sample preparation; the evaluation of the junction parameters can be found in the Methods: Sample characterization. In the experiment, magnetic Co/Cr MFM tip is scanned over the device and probes its local magnetic properties. Concomitantly, it induces a local highly inhomogeneous oscillating magnetic field that affects the dynamics of Josephson vortices inside JJ. The local response is revealed in MFM maps; the global response of the device is probed by measuring transport properties of the junction as a function of tip position, external magnetic field and bias current through the junction (see Methods: sample characterization). Simultaneously, we detect the reverse action of the Josephson vortex dynamics, triggered by the oscillating tip, on the phase and the amplitude of tip oscillations. A comprehensive analysis of the mutual action and counteraction between the tip and the device, along with supporting numerical modeling, enables an unambiguous identification of peculiarities of the Josephson vortex dynamics in the device. The demonstration of a local generation, detection, and manipulation of Josephson vortex is the main result of our work.

## Results

### Global and local magnetic responses of the device

Figure 1b shows *I*_c_ (*H*_ext_) dependence measured in the external magnetic field *H*_ext_ applied perpendicular to the junction plane; the tip was retracted far away from the device. The junction exhibits a regular symmetric Fraunhofer-type *I*_c_ (*H*_ext_) pattern, indicating a good uniformity of the junction. The central lobe of *I*_c_ (*H*_ext_) is significantly wider than the side lobes and decays quasi-linearly with increasing *H*_ext_. This is a well known fingerprint of a long JJ^15^, which length (*L* = 2500 nm in our device) is significantly larger than the effective Josephson penetration depth of the JJ *λ*_J_. The estimations of characteristic junction parameters (see Methods: Sample characterization) indicate that our JJ is moderately long *L* ≃ (5 ÷ 7)*λ*_J_, consistent with the quasi-linear shape of the central *I*_c_ (*H*_ext_) lobe.

**Fig. 2:**
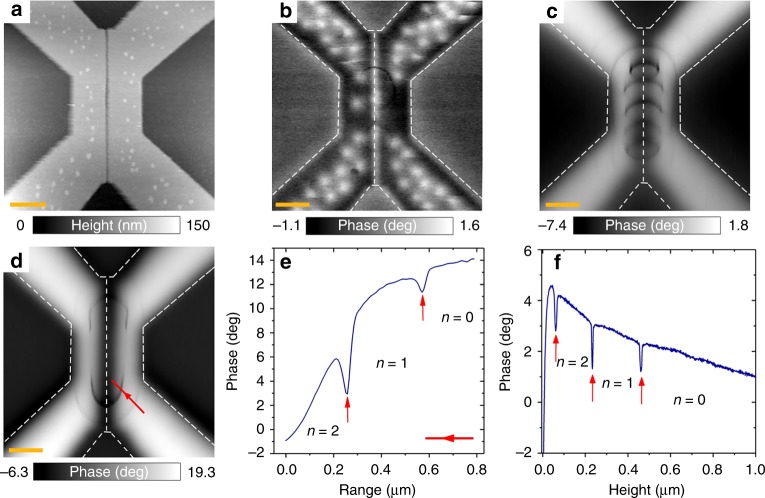
Detection of Josephson vortices. **a** Topographic AFM image of the device. The orange scale bar corresponds to 0.5 μm. (**a**–**c**) MFM phase maps (dashed lines represent the edges of the device): **b** when the device is field-cooled in 90 Oe (tip lifted by 150 nm). The areas with screened (enhanced) field appear in black (white); small round white spots are individual Abrikosov vortices pinned in Nb. **c** when a 90 Oe field is applied to the zero-field cooled device (tip lifted by 70 nm). Meissner currents screen the magnetic field in Nb; no Abrikosov vortex are present. Several black rings appear near the junction area representing sharp phase drops occurring when the tip is positioned in specific locations. The rings delimit regions of specific Josephson vortex configurations inside the junction affected by the local magnetic field of the tip (see in the text). **d** when no field is applied to zero-field cooled device (tip lifted by 70 nm). A few black arcs are visible, demonstrating the effect of the self-field of the magnetic tip on the junction. **e** spatial variation of the phase signal along the line represented by the red arrow on the map **d**. Each phase drop (vertical red arrows) delimits different Josephson configurations with the vortex numbers *n* = 0, 1, 2 (see in the text). **f** evolution of the phase as a function of the tip height (tip-surface distance) when the tip is positioned above the center of the device. Red arrows and vortex numbers *n* = 0, 1, 2—the same as in **e**

Figure 2a presents the topographic AFM image of the same junction (see Methods: AFM and MFM experiment).

The two Nb-electrodes of the device appear in light gray, the junction region—a dark-gray slit in the image center. In Fig. 2b–d we show a series of MFM maps of the device. There the gray contrast encodes the locally measured phase of the cantilever oscillations; the phase shift is very sensitive both to the gradient of magnetic force acting on the tip^24,46^ and to the dissipation (see Methods: Dissipation and phase shift in MFM). Figure 2b presents the magnetic map of the device field-cooled at *H*_ext_ = 90 Oe. Here bright spots represent individual Abrikosov vortices firmly pinned in the superconducting Nb leads. Meissner currents circulating at the edges of the device produce an additional white-black contrast.

Figure 2c, d show the magnetic map of zero-field cooled device. In Fig. 2c the field *H*_ext_ = 90 Oe was applied at low temperature prior to imaging; in Fig. 2d no field was applied. In these maps the Nb leads remain in the Meissner state, Abrikosov vortices do not penetrate. On both maps, the striking features are large concentric black rings and arcs surrounding the junction area. In addition, at finite external fields, Fig. 2c, there are also smaller black rings visible in the middle of the junction, forming a chain. Thus both the external field and the magnetic field of the tip play essential roles in the phenomenon. The evolution of the ring patterns with in the applied field can be seen in the Methods: mechanism of detection of Josephson vortices by MFM.

### Generation of Josephson vortices

The observed rings/arcs are puzzling. First, they are symmetric with respect to the Josephson junction (vertical) axis, and also, they are almost symmetric with respect to the horizontal axis of symmetry of the device. Second, all rings appear in black on the maps, they correspond to sudden phase drops, as confirmed by the cross-section plot in Fig. 2e. Third, the rings/arcs located close to the junction are characterized by a higher amplitude than the distant ones (compare the minima marked by red arrows in Fig. 2e). Fourth, the section of the rings/arcs in the radial direction is very small, ~5−2 nm, or even smaller, often limited to a single pixel on the image. This is much shorter than both Josephson *λ*_J_ ~ 400 nm and London *λ*_Nb_ ~80 nm penetration depths of the device, the scale on which the magnetic features are expected to spatially evolve, as it is indeed the case with the Abrikosov vortex observed in Fig. 2b. Moreover, the tip being located quite far from the device (70–150 nm), there is a priori no reason to expect so sharp variations.

To understand the origin of the phenomenon, we provided an additional experiment in which the tip was initially placed above the device center 1 μm away from the surface, and then moved towards the device. The evolution of the phase with the tip height is presented in Fig. 2f. As the tip is approached, the general trend is a smooth phase increase. This is expected: The tip-device interaction is a repulsion due to the supercurrents circulating across the junction to screen the magnetic field of the tip (the same diamagnetic repulsion makes magnets levitate above superconductors). As the tip gets closer the screening currents and the resulting repulsion force gradient increase^47^. The latter provokes a shift in the phase of the oscillations, measured at a fixed frequency (see Methods: Dissipation and phase shift in MFM).

A smooth increase of the phase signal in Fig. 2f is interrupted by a series of three sharp phase drops. We suggest the phenomenon to happen when the oscillating tip triggers Josephson vortices penetrations/exits to/from the junction. Indeed, in our experiments the magnetic tip is situated above the device.As a response to this magnetic perturbation, there are always screening currents crossing the JJ. The Josephson vortex motion inside the junction perturbs the screening current flow across it. The screening efficiency reduces, the resonance frequency decreases and the dissipation due to the Josephson vortex motion rises, resulting in a phase signal drop. Additional experiments confirmed that observed phase drops correspond indeed to both cantilever frequency shifts and dissipation (see Methods: Dissipation and phase shift in MFM). It becomes immediately clear why the drops have a larger amplitude when the tip is positioned closer to the JJ area (Fig. 2c–e). There the oscillatory motion of the tip becomes very sensitive to what happens inside the junction.

## Discussion

### Evolution of phase signal with respect to tip-JJ distance

We can now qualitatively understand the evolution of the phase signal measured during the tip approach, Fig. 2f. When the tip is far from the JJ, there are no Josephson vortices inside, the device is in a *n* = 0 state. As the tip is approached, the total energy of this *n* = 0 state rapidly increases due to both, the kinetic energy of screening currents and the current-generated magnetic energy. We suggest that the first phase drop occurs when the rising energy of the *n* = 0 state equals the energy of the state with one Josephson vortex inside the device, that is *n* = 1. This occurs at ~ 450 nm. At this specific height the oscillating field of the tip provokes rapid entry/exists of the first Josephson vortex into/from the junction, resulting in the phase drop. Just below 450 nm the *n* = 1 state is thermodynamically stable. However, when the tip is further approached, the energy of this state increases; a new phase drop occurs at ~220 nm. At this position of the tip the system oscillates between *n* = 1 and *n* = 2 states. The transition to the *n*=3 state occurs at the tip height ~ 50 nm. The same phenomena take place when the tip moves laterally at a fixed height (Fig. 2c, e). Thus, the phase drops occur at positions of the tip in space at which the systems oscillates between the two neighboring Josephson vortex configurations: 0 ⇔ 1, 1 ⇔ 2, and 2 ⇔ 3 in Fig. 2f, 0 ⇔ 1, and 1 ⇔ 2 in Fig. 2e. Therefore, the phase drops delimit the regions characterized by a fixed number of Josephson vortices, *n* = 0, 1, 2, 3…

The direct link between the phase drops and Josephson vortices is further confirmed by the MFM map of the junction subject to the external magnetic field *H*_ext_ = 90 Oe, Fig. 2c. This map contains, in addition to large coaxial rings of Fig. 2d (*H*_ext_ = 0), a series of small rings forming a chain along the junction. Understanding the origin of these additional rings is straightforward, since in this case, the junction already contains a chain of field-induced Josephson vortices, even if the MFM tip is absent. The number of vortices in the chain corresponds to the number of lobes in *I*_c_ (*H*) modulation, minus the central one, which represents the Meissner state. The examination of *I*_c_ (*H*) pattern from Fig. 1 suggests that at 90 Oe the junction contains a chain of seven Josephson vortices. The vortex chain creates inhomogeneous magnetic field distribution in the junction with a finite field gradient (as can be seen from Supplementary Movie 3). Upon scanning, the tip interacts with the Josephson vortex chain, leading to an extra signal. The comparison between zero-field map Fig. 2d and 90 Oe map, Fig. 2c suggests that the large concentric rings represent Josephson vortices induced solely by the tip field *H*_tip_, whereas small rings reflect the interaction of the tip with the vortex chain. Remarkably, only five small rings are visible in Fig. 2c, instead of seven expected. The reason is that two vortices are pushed out of the junction by the tip field, since in this experiment *H*_ext_ and *H*_tip_ were oppositely directed. Thus, the MFM tip is also able to modify the number of Josephson vortices initially present in the junction. This enables a local control of the global response of the device, as we demonstrate later.

### Modeling and simulations

**Fig. 3:**
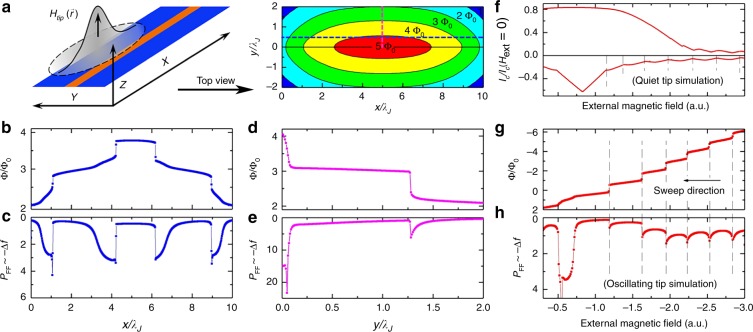
Modeling the experiment. **a** A sketch of the junction and the local field induced by the tip; top view of magnetic flux in the junction upon scanning at *H*_ext_ = 0. Black contour lines represent tip positions at which the number of flux quanta changes. These lines are the bifurcation points for entrance/exit of a *n* ± 1 Josephson vortex (see in the text). The similarity with observed black rings in Fig. 2d is noticeable. **b**–**e** Simulated junction responses at *H*_ext_ = 0 for tip scans along (**b**, **c**) and across (**d**, **e**) the junction, following blue and pink dashed lines in **a**. **f**–**h** simulation of the Josephson vortex penetration upon the field scans with a tip located close to the junction edge (*x* = 0.1; *y* = 0). **f** field-dependence of the critical current at oppositely directed external fields *H*_ext_ shows asymmetric behavior due to the additional flux from the tip. Panels **b**, **d**, **g** show the total flux in the junction. Steps represent abrupt entrance/exit of Josephson vortices. Panels **c**, **e**, **h** show the energy losses *P*_FF_ in the junction, due to the Josephson vortex flux-flow induced by the oscillating tip. The dissipation peaks, similar to those observed in the experiment, occur at the bifurcation points for entrance/exit of a *n* ± 1 Josephson vortex (see in the text)

A deeper insight is brought by the numerical modeling of the junction dynamics in the presence of MFM tip; the results are presented in Fig. 3 (see also Methods: Numerical modeling). In our simulation, Fig. 3a, the device is in the (*x*, *y*) plane, and the JJ is represented by a horizontal line with coordinates (*x*, *y*, *z*) (0, 0, 0) − (10, 0, 0) (all coordinates are normalized by *λ*_J_). A zero-external field is considered. The MFM tip introduces a spatially non-uniform magnetic field, which affects the total flux crossing the junction. The induced flux depends on the position of the tip and attends its maximum when the tip is placed above the center of the junction, right panel in Fig. 3a. Contour lines represent the tip positions at which an integer number of flux quanta are induced, at zero-external field and current (*H*_ext_ = 0; *I* = 0). They correspond to the expected bifurcation points for entrance/exit of an extra Josephson vortex, *n* ⇔ *n*±1. A qualitative similarity with the reported “rings” in Fig. 2d is obvious. In Fig. 3b, c we plot, respectively, the evolution of the magnetic flux and the dissipation *P*_FF_ in the junction as a function of the position of the MFM tip moving along the *x*-direction at *y* = 0.5 (horizontal dashed blue line in Fig. 3a). Figure 3d, e display the same information for the tip moving in the *y*-direction at *x* = 5 (vertical dashed pink line in Fig. 3a). The first important conclusion here is that the tip indeed generates Josephson vortices even at zero applied field/current. The generation process strongly depends on the tip location.

The second important result of the simulation is a series of jumps in the energy losses of the JJ which occur each time a new Josephson vortex enters to (or exists from) the junction, Fig. 3c, e (see also Methods: mechanism of detection of Josephson vortices by MFM). These peaks are very similar to those observed in the experiment, Fig. 2e, f. The peaks occur at the bifurcation points, and are related to a dynamic perturbation of the JJ caused by the tip oscillations in the *z*-direction. This dynamic perturbation is weak and does not depin Abrikosov vortices (Fig. 2b). Though, it does affect the motion Josephson vortices which become very mobile near the bifurcation points. Indeed, the only pining they experience is the surface pinning at the junction edges. The critical current in a long junction can be considered as a depining current through such a surface barrier. At the bifurcation points the critical current of the junction is reduced, see Fig. 3f, suppressing the pinning. As a result, at the bifurcation points even a very small modulation of the tip field triggers entrance/exit and in/out motion of Josephson vortices. In SNS junctions it leads to the appearance of a time-dependent voltage and consequent flux-flow losses (more details see in Methods: Mechanism of detection of Josephson vortices by MFM). Figure 3c, e show the energy losses in the junction induced solely by the tip. Correlations with the flux jumps in Fig. 3b, d demonstrate that the flux-flow losses are indeed at maximum at the bifurcation points. Simulations clarify the abruptness of observed MFM phase drops. Essentially, the small oscillation amplitude of the tip can trigger significant vortex motion only in a narrow range of parameters (tip position or external field/current) close to bifurcation points. The corresponding energy exchange between the tip and the device leads to an additional damping of MFM oscillations, which is detected as a phase shift in our experiment (see Methods: dissipation and phase shift in MFM). A good qualitative similarity between the experimental data in Fig. 2b and numerical simulations in Fig. 3a, as well as between Figs. 2e and 3e, support our conclusions and confirm that it is indeed possible to manipulate Josephson vortices by the MFM tip.

### Locally influencing the global response of the device

**Fig. 4:**
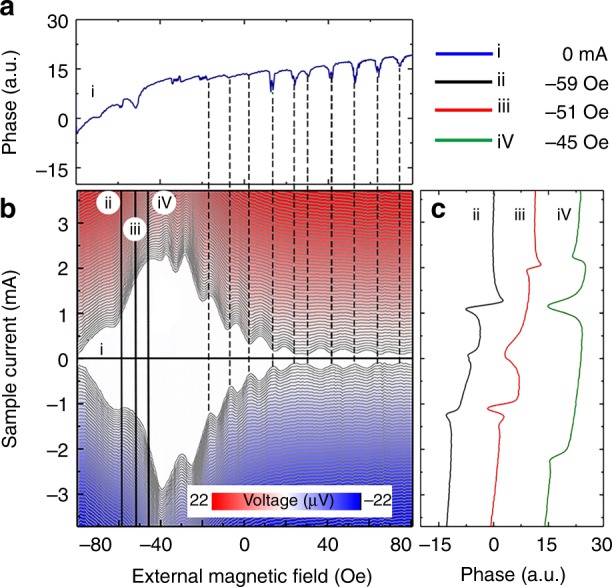
Electronic properties of the device in the presence of the MFM tip 70 nm above the bottom edge of the junction. **a** Color-coded plot: the voltage drop across the junction measured as a function of applied current and external magnetic field. Red (blue): positive (negative) voltage drop; white: zero-voltage drop (superconducting regions). **b** phase shift of the cantilever at zero current, corresponding to the cross-section (i) of the Fraunhofer pattern. Vertical dashed lines show correlations between the phase and the critical current. **c** phase vs current recorded at the magnetic fields −59 Oe (black curve), −51.4 Oe (red curve), and −45.6 Oe (green curve). The curves correspond to the cross-sections (ii), (iii), and (iv) respectively of the Fraunhofer pattern. The phase drops positions correlate with the critical current

We now demonstrate the effect of the magnetic tip on the magneto-transport properties of the device. In this experiment the tip was positioned above the bottom edge of the Josephson junction, and the measurements (as described before, Fig. 1b) were performed; the phase evolution was recorded simultaneously. The result is presented in Fig. 4a–c. The first effect there is a strong asymmetry of the Fraunhofer pattern (as compared with that measured without magnetic tip, Fig. 1b): The maximum critical current is obtained when an external field of about −40 Oe is applied. To understand the effect we remind that the total magnetic field *H*_total_(*r*) at the location *r* of the device is the sum of the externally applied field *H*_ext_ and a spatially inhomogeneous stray field of the tip *H*_tip_(*r*). Furthermore, the maximum critical current should correspond to *H*_total_ ≃ 0, i.e. *H*_ext_ ≃ −*H*_tip_. It means that the tip situated 70 nm away from the device produces at the junction a field ~40 Oe. The second effect is a significantly weaker contrast and distortions in the Fraunhofer pattern, as compared to the case displayed in Fig. 1b. This may come from the spatial inhomogeneity of *H*_tip_. The third effect is the critical current asymmetry with respect to the direction of the transport current. The asymmetry can be due to a non-uniform distribution of the total current density which is the sum of the transport, Meissner and Josephson currents.

Further, the phase evolution measured at zero-current as a function of the applied field is presented on Fig. 4a. It is clear, that the phase drops coincide with Fraunhofer oscillations, thus confirming the general scenario of the effect that we suggested above. It should be noted that at zero-current the DC-transport experiments provide no information about the state of the junction while the phase of the tip oscillations does.

The results of numerical simulations of this experiment are presented in Fig. 3f–h. Here the external field was swept from a finite value to zero; the tip was considered located close to the JJ edge, *x* = 0.1, *y* = 0. Figure 3f shows the critical current *I*_c_(*H*) modulation pattern, which is strongly distorted by the presence of the tip, as experimentally observed. As the field is reduced, the sequential exit of Josephson vortices causes the dissipation similar to the experimentally observed ones (Fig. 4a). Note a slight offset between cusps in Fig. 3f–h, caused by different simulation conditions. In Fig. 3f the tip was supposed still, while it was considered oscillating in Fig. 3g, h. The corresponding variations of *H*_tip_ lead to small yet observable shifts of *H*_ext_ at which entrances/exits of vortices occur (vertical dashed lines in Fig. 3f–h). This simulation also suggests that the number of accessible Josephson states can be extended by applying an external field; in this way we were indeed able to experimentally generate Josephson states up to *n* = 10. In principle, the device could accept a yet larger number of Josephson vortices, ~*L*/*ξ*_Nb_ ≃ 30, the number being limited by the superconducting phase gradients reaching ∇*φ* ~ *π*/*ξ*_Nb_ at critical values of screening currents in Nb-electrodes. Notice that the numerical simulations nicely reproduce all essential experimental observations, thus confirming the correctness of the suggested microscopic physical model of the phenomenon.

Finally, we measured the phase vs transport current relation at three different magnetic fields (Fig. 4c). While the main phase drops predictably occur close to the positive and negatives critical current values, additional features are observed at lower current values, probably reflecting local rearrangements of Josephson vortex inside the junction at a fixed *n*. Clearly, the phase signal contains a more rich information about the JJ as compared to the conventional DC-transport.

In conclusion, we demonstrated a way of a remote generation, detection and manipulation of Josephson vortices inside planar Josephson junctions, using a low temperature MFM. Local MFM experiments were combined with simultaneous DC-transport measurements. Our main result is the observation of a singular response of the MFM tip at specific set of parameters (tip location, temperature, external field and currents), which results in sharp rings/arcs in MFM maps, due to phase drops in the cantilever oscillation. These singularities are identified as bifurcation points between neighboring Josephson states characterized by different number/position of Josephson vortices inside the junction. We developed a model that strongly supports our findings. It confirms the importance of the tip-device energy exchange at the bifurcation points and demonstrates that MFM can provide a unique information about the Josephson vortex state, significantly richer than conventional transport measurements. The MFM tip can trigger and detect Josephson vortex motion in the junction without a need for transport current or external magnetic field and, therefore, can be used as a local probe of Josephson vortex dynamics. We anticipate that our finding will boost the development of new MFM-based methods of a local non-contact inspection and control of advanced superconducting quantum electronics devices.

## Methods

### AFM and MFM experiment

The experiments were carried out on AttoCube scanning probe system (AttoDry 1000/SU) at temperatures ranging from 4 K to 12 K and in the external magnetic field up to 200 Oe (Fig. 1). The device topography (Fig. 2a) and its magnetic response (Fig. 2b–d) were studied using a standard magnetic Co/Cr-coated cantilever (MESP, Bruker, 2.8 N/m spring constant). In the experiments, the cantilever with the tip is excited by a dither. The amplitude and the phase of the cantilever oscillations is measured at a fixed resonance frequency, typically 87 kHz, corresponding to the resonance of the cantilever in the absence of tip-device interactions. Since the phase signal strongly varies at the resonance, it is very sensitive to tiny frequency shifts.

### Sample preparation

Nb/Cu/Nb SNS structures were fabricated using UHV magnetron sputtering, e-beam lithography technique with hard mask, and plasma-chemical etching as follows. First, a 50-nm Cu film and 100 nm Nb film were subsequently deposited onto SiO_2_/Si substrate in a single vacuum cycle^48^. The polymer mask for Nb leads was then formed by electron lithography. The pattern was covered by a 20-nm-thick aluminum layer lifted off, the Al hard mask for Nb leads was formed. Next, uncovered Nb was etched by the plasma-chemical process. After Nb patterning the Al mask was removed with wet chemistry. The resulted device studied in this work has the following geometrical characteristics: the junction length is 2500 nm, its width is 200 nm, the width of Nb leads in the JJ area is 500 nm (see Fig. 2a).

### Sample characterization

The electron transport measurements were made in a standard four-terminal configuration. To examine basic current-field characteristics of the device, the magnetic tip was retracted far away from the sample to exclude the influence of its stray magnetic field. The critical temperature of the superconducting junction in zero applied filed was 7.2 K, the critical current at 4.2 K was 2.8 mA.

The junction presented in the main text has the following parameters at the corresponding operation temperature: the length *L* = 2.5 μm, the width of the Cu interlayer *t*_N_ = 200 nm, the thickness of Cu interlayer *d*_N_ = 50 nm, the width of each Nb electrode *W*_S1_ ≃ *W*_S2_ ≃ 500 nm, the thickness of Nb electrodes *d*_S_ = 100 nm, the London penetration depth of Nb electrodes *λ*_S_ ≃ 80 nm, the Josephson critical current *I*_c_ ≃ 3 mA, and the critical current density *J*_c_ = *I*_c_/*Ld*_N_ ≃ 2.4 × 10^6^ A cm^−2^.

Our junctions have planar geometry *W*_S1_ + *W*_S2_ ≫ *d*_S_. Such junctions are different from conventional overlap (sandwich) type junctions in two respects: (i) Planar junctions have significant demagnetization factor *n* ~ 1 because the field is applied perpendicular to thin film superconducting electrodes. This leads to flux focusing effect^49^, due to which the effective magnetic field in the junction is larger than the applied field by the factor (1 − *n*)^−1^≫1. (ii) The perpendicular to the electrodes magnetic field is screened and spread out along surfaces of the electrodes. Thus, screening Meissner currents are generated over the whole area of the electrodes, and not just in a thin layer ~*λ*_S_ adjacent to the junction. This leads to non-locality of electrodynamics in planar junctions with thin electrodes *d*_S_ < *λ*_S_^50–52^.

The two mentioned peculiarities lead to principle modification of the effective magnetic width of the junction *W*_eff_, which determines the relation between the flux in the junction, Φ, and the applied field, *H*, *W*_eff_ = Φ/*LH*. For elongated planar junctions with the widths of the two electrodes *W*_S1,2_ < *L*, as in our case, magnetic flux from half the width of each electrode enters the junction^51^. The physical origin of this is quite simple. Perpendicular to electrodes magnetic field is spread evenly along the surface of the electrode so that approximately half of the flux within the electrode area is guided into the junction^49^. For the studied junction *W*_eff_ ≃ *t*_*N*_ + (*W*_S1_ + *W*_S2_)/2 ≃ 700 nm.

The corresponding flux quantization field is ${\mathrm{\Delta }}H \simeq \frac{{{\mathrm{\Phi }}_0}}{{LW_{{\mathrm{eff}}}}} \simeq 11.8\,{\mathrm{Oe}}$, which is only slightly larger than experimentally observed value Δ*H* ≃ 10 Oe, see Fig. 1b. Most likely this is due to expansion of electrode widths at the ends of the junctions, see Fig. 2a, which leads to a slightly larger average magnetic width *W*_eff_ ≃ 830 nm.

For a conventional overlap (local) junction the Josephson penetration depth is $\lambda _{\mathrm{J}} = \sqrt {\frac{{{\mathrm{\Phi }}_0c}}{{8\pi ^2{\mathrm{\Lambda }}J_{\mathrm{c}}}}}$, where Λ = *t*_*N*_ + *λ*_S1_ + *λ*_S2_ is the magnetic thickness of the junction and *λ*_S1,2_ are London penetration depths of the two electrodes.

Estimation of the Josephson penetration depth in our planar junctions is more complicated. Namely, unlike overlap junctions, Josephson vortex shape in a planar junction is not described by a single length scale^50^. Instead, the central strongly non-linear “core” region is characterized by the length $\lambda _{\mathrm{J}}(0) = \frac{{\lambda _{\mathrm{J}}^2}}{{\lambda _{\mathrm{S}}}}$. But the tail of the vortex is decaying non-exponentially with the characteristic length scale $\lambda _J(\infty ) = \lambda _J(0)\frac{{2\lambda _{\mathrm{S}}}}{{d_{\mathrm{S}}}}$.

For the studied junction we obtain: *λ*_J_(0) ≃ 380 nm and *λ*_J_(∞) ≃ 220 nm. More accurate estimation of the Josephson penetration depth in the studied junction is complicated by the lack of accurate analytic expression for the intermediate case *d*_S_ ≃ *λ*_S_ between local and non-local electrodynamics^52^. Another way of estimation of the effective Josephson length *λ*_Jeff_ by analyzing the lower critical field for penetration field of the first Josephson vortex, *H*_c1_. It corresponds to the end of the linear central lobe of *I*_c_ (*H*).

Taking the standard expression *H*_c1_ = 2Φ_0_/*π*^2^*λ*_Jeff_*W*_eff_ and using *W*_eff_ = Φ_0_/*L*Δ*H*, we obtain *λ*_Jeff_ ≃ (Δ*H*/*H*_c1_)2*L*/*π*^2^, which also gives a value close to *λ*_*J*_(0). We conclude that the effective Josephson penetration depth of our junction is significantly smaller than the junction length. Therefore, our junction is moderately long *L*/*λ*_Jeff_ ~5–7. This is consistent with presence of the linear central lobe of *I*_c_(*H*) pattern, see Fig. 1b, representing the screened Meissner state without vortices in the junction^53^.

### Numerical modeling

To model the behavior of the junction in the presence of the MFM tip we solve the sine-Gordon equation for the time and space dependence of the Josephson phase difference in the junction *φ* (*t*, *x*):

1 $$\varphi \prime\prime - \ddot \varphi - \alpha \dot \varphi = {\mathrm{sin}}\varphi - \gamma ,$$

ith the boundary conditions at the junction edges

2 $$\varphi \prime = \frac{{2\pi {\mathrm{\Lambda }}}}{{{\mathrm{\Phi }}_0}}H(x).$$

Here primes and dots denote spatial and time derivatives, respectively, *α* is the quasiparticle damping parameter and *γ* = *I*/*I*_c_ is the normalized bias current. Space and time are normalized by the Josephson penetration length and inverse Josephson plasma frequency $\omega _{\mathrm{p}}^{ - 1}$, respectively. The field is normalized by *H*_0_ = Φ_0_/2*π*Λ*λ*_J_ = (*π*/4)*H*_c1_. Details of the formalism can be found e.g. in ref. ^53,54^. We assume that the junction line has coordinates (*x*, *y*, *z*) (0, 0, 0) − (*L*, 0, 0) with electrodes in the (*x*, *y*) plane, as sketched in Fig. 3a.

The MFM tip introduces spatially non-uniform magnetic field in the junction *H*_tip_(*x*). It enters the boundary conditions, Eq. (2), and generates the tip-induced phase shift within the junction, corresponding to the integral of Eq. (2). The MFM tip has a conical shape with a few micron broad base and a sharp end ~30 nm. Therefore, we model the tip field by two Gaussian peaks: a broad and a narrow, representing the tip base and the tip end, correspondingly. Additional information can be found in the Supplementary Fig. 1. We have been trying a variety of different parameters of the tip and relative junction lengths aiming at qualitative clarification of the observed phenomena. Although shapes of characteristics do depend on junction and tip parameters, qualitative results remained the same.

In the dynamic case the tip is oscillating harmonically with a small amplitude and at a frequency much smaller than *ω*_*p*_ (about 80 kHz, compared to *ω*_*p*_/2*π* > 10 GHz). The induced field in the junction, however, may be unharmonic in time due to rapidly decaying field from a sharp end of the tip. In our simulations we tested both harmonic and anharmonic tip fields. There was no significant difference between those cases. The dynamic results presented in Fig. 4c–i are obtained for the unharmonic tip field, proportional to 1 + *a*[0.5(1−cos (*ωt*))]^3^. To speed up calculation the tip angular frequency was set to *ω* = 0.05*ω*_*p*_ and the total integration time was 20*π*/*ω*. It was checked that changing *ω* by factor two in both directions does not affect the results. The quasiparticle damping factor was set to *α* = 1, to get overdamped phase dynamics typical for SNS junctions.

Blue symbols in the Supplementary Fig. 1 represent simulated *I*_c_(*H*) patterns for a long junction *L*/*λ*_J_ = 10 without a tip (*x* = ∞). Here one can clearly see the central Meissner lobe, ending at *H*_c1_, followed by smaller lobes associated with incremental entrance/exit of one Josephson vortex. Red symbols in the Supplementary Fig. 1 represent *I*_c_(*H*) patterns with a static tip placed close to the left edge of the junction *x* = 0.1*λ*_J_, *y* = 0, *z* = 0. The tip field is described by the broad Gaussian with the width *σ*_1_ = 5*λ*_J_ = *L*/2 and the total flux Φ_1_ = 5Φ_0_ and a narrow one with *σ*_2_  = 0.1*λ*_J_ and Φ_2_ = 0.5Φ_0_. It is seen that the positive field of the tip leads to displacement of the middle point of the central lobe, corresponding to Φ = 0, to a negative field. Furthermore, the *I*_c_ (*H*) is distorted so that positive and negative currents become dissimilar. This type of asymmetry is also seen in the experimental curve in Fig. 4. It is a consequence of removal of the space (left-right) symmetry by the tip^53^. Such the asymmetry in caused by the spatial non-uniformity of the tip field. Since the Lorentz force on JVs depends on the sign of the bias current, positive and negative currents persuade JV entrance from opposite junction sides. The non-uniform tip field creates different boundary conditions for JV entrance at the two edges of the JJ and, therefore, leads to dissimilar positive and negative *I*_c_.

We want to emphasize that in long junctions vortex states are metastable, i.e., for a given field the junction may have either *n* or *n* + 1 Josephson vortices. The metastability is most pronounced at bifurcation points between nearby *I*_c_(*H*) lobes at which *n* and *n* + 1 vortex states are degenerate. Away from those bifurcation points the system may stay for a while in the initial metastable state despite a higher energy. This leads to history dependent hysteresis. Such metastability can be seen as points below the envelope with maximal *I*_c_ (*H*) in the Supplementary Fig. 1. Due to quantized nature of vortices, the transition between adjacent *n*/*n* + 1 states is abrupt. Even though this may not be well seen in *I*_c_, it is quite pronounced in the step-like change of flux in the junction, shown in Fig. 3c, e, h. The metastability and abrupt switching between *n*/*n* + 1 Josephson vortex states leads to the abrupt response of the MFM tip.

When the tip is moving along the electrodes, the field and the flux induced by the tip in the junction is changing. For example, when the tip is placed at the edges of the junction (*x*, *y*, *z*) = (0, 0, *z*) or (*L*, 0, z), only half of the total flux penetrates the junction. However, when the tip is in the middle of the junction (*L*/2, 0,z) almost all the flux penetrates in the junction, provided the tip field is narrower than the junction *σ*_1,2_ < *L*/2. This is clearly seen in Fig. 3c, which shows the total flux in the junction upon scanning of the tip along the line *y* = 0.5 parallel to the junction at *H* = 0. It is seen that when the tip is at the edge *x* = 0 there are two Josephson vortices Φ = 2Φ_0_. Upon moving of the tip inside, the third Josephson vortex jumps in at *x* ≃ 1 and finally the fourth at *x* ≃ 4 when the tip is approaching the middle of the junction. Note that despite integer number of Josephson vortices, the total flux is not perfectly quantized. This is due to the finite length of the junction, leading to incomplete screening (confinement) of the vortex field.

Similarly, the field of the tip is increased by factor two when the tip is moving towards the center *x* = 5, *y* = 0 in the *y* direction. As shown in Fig. 3e, the induced flux is increased from 2Φ_0_ at the edge *y* = 2 to 4Φ_0_ in the center *y* = 0.

Thus, the induced flux is at maximum when the tip is placed in the center of the junction (*L*/2, 0, 0). Moving away from the center in any direction leads to reduction of the tip field and flux. Figure 3b represents calculated induced (unscreened) flux in the junction at *H* = 0 upon scanning of the tip in the (*x*, *y*) plane.

Contour lines represents tip positions at which integer flux quanta are induced, corresponding to expected bifurcation points for entrance/exit of a Josephson vortex. A qualitative similarity with the reported”rings and arcs” in Fig. 2d is obvious. Note that the actual number of vortices in the junction, obtained by solving the sine-Gordon Eq. (1), see Fig. 3c, e, is smaller by ~1 compared to the ratio of the induced (applied) flux to Φ_0_. This is due to finite screening of the field by the long junction.

### Mechanism of detection of Josephson vortices by MFM

The shift of the central lobe of *I*_c_(*H*) patterns upon engagement of the MFM tip, c.f. Figs, 1b and 4, and Supplementary Fig. 1, indicates that the tip generates a significant static flux in the junction, sufficient for introduction of several Josephson vortices. The dynamic perturbation by the oscillating tip is small. For example, it does not depin Abrikosov vortices, as seen from clear images in Fig. 2b. However, Josephson vortices are much more mobile. The only pining they experience is the surface pinning due to interaction of the Josephson vortex with its image antivortex at the edges of the junction. The critical current in a long junction can be considered as a depining current through such a surface barrier. The MFM tip amplitude in this experiment was kept small in order not to disturb radically the static vortex arrangement so that *I*_c_ (*H*) modulation patterns with static and oscillating tips are similar. However, at integer flux quanta in the junction the critical current becomes very small (vanishes), see Figs. 1b and 4. Those points represent bifurcation point with equal energies for *n* and *n* + 1 fluxons in the junction. Since at those points *I*_c_ ~ 0, the surface pinning is very small and even a very small oscillating field from the tip removes the degeneracy between *n* and *n* + 1 states and thus introduces (*n* → *n* + 1) or removes (*n* + 1 → *n*) one Josephson vortex. Once an extra vortex is introduced, it is no longer pinned by the edge and can move freely in the junction. Most commonly the extra vortex will shuttle back and force near the edge. However numerical simulations have demonstrated that at bifurcation points a ratchet-like rectification phenomenon^53,55^ often takes place leading to unidirectional vortex motion. Different types of tip-induced Josephson vortex dynamics can be seen in provided Supplementary Movies 1–3 and they description in Supplementary Fig. 2.

Tip-induced Josephson vortex motion leads to appearance of flux-flow voltage according to the ac-Josephson relation *V*_FF_ = (Φ_0_/2*π*)*dφ*/*dt* and, consequently, to dissipation of energy in the junction $P_{{\mathrm{FF}}} = V_{{\mathrm{FF}}}^2/R_{\mathrm{n}}$, where *R*_n_ is the junction normal (quasiparticle) resistance. This is the main mechanism of interaction and exchange of energy between the MFM tip and the junction. This energy transfer leads to damping of tip oscillations, which leads to the phase shift measured in experiment.

Essentially, the oscillating MFM tip triggers entrance/exit and motion of Josephson vortices, which leads to flux-flow losses in the junction. The corresponding energy exchange between the tip and the junction leads to additional damping of MFM oscillations, which is detected as a phase shift in our experiment. However, such tip-induced Josephson vortex motion occurs only at the bifurcation points close to entrance/exit of one vortex. This explains the abrupt nature and a very narrow range of parameters (tip position, external field or bias current) at which the energy exchange between the tip and moving Josephson vortices occurs. A good qualitative similarity between experimental data and numerical simulations support our conclusions, compare Figs. 2d and 3b–d, Figs. 2e and 3f, and Figs. 4 and 3i. Numerical simulations confirmed that it is indeed possible to manipulate Josephson vortices by the tip at the bifurcation points. For example, it is possible to organize ratchet-like rectified vortex motion^53,55^. The corresponding dc voltages at *ω* ~ 100 kHz are in the sub nV range and could be detected directly using a SQUID voltmeter.

To visualize the correlation between MFM experimental data and simulation data, we present additional movies.

Supplementary Movie files 1–3 show simulated junction dynamics during one period of tip oscillations. Supplementary Fig. 2 shows one frame of the video with clarifying comments. There are three panels in the video and parameters. The top panel shows spatial variation of the supercurrent density $J_{\mathrm{s}}(x)/J_{\mathrm{c}} = {\mathrm{sin}}(\varphi )$. The vortex is seen as an up(+1)-down(−1) variation of ${\mathrm{sin}}(\varphi )$ with zero in the center of the vortex. Horizontal grid spacing is 0.5 and vertical grid spacing is 2*λ*_j_ (in all panels). The middle panel shows spatial distribution of voltage $V(x) \propto d\varphi (x)/dt$. The total vertical scale is equal to the plasma voltage from −*V*_p_ to $+ V_{\mathrm{p}} = \frac{{{\mathrm{\Phi }}_0}}{{2\pi }}\omega _{\mathrm{p}}$. The entering vortex leads to appearance of a negative voltage peak marked in the Figure (positive vortex moving in positive direction generates negative voltage because $d {\varphi} / {dt} < 0$). From the maximum amplitude of the voltage it follows that the characteristic jump-in time of the vortex is several plasma periods $2\pi /\omega _{\mathrm{p}}$, independent of the tip frequency. The bottom panel shows spatial distribution of magnetic induction in the junction $B(x) \propto d\varphi (x)/dx$. The horizontal grid spacing is $3.(3)H_0 = 3.(3){\mathrm{\Phi }}_0/2\pi {\mathrm{\Lambda }}\lambda _{\mathrm{J}}$. The lowest gridline corresponds to the applied field *H*. Therefore, in this panel only contribution from the tip and from Josephson vortices are seen. The inhomogeneous field of the tip, located at the left side of the junction *x* = 0.1, is changing periodically with time. From Supplementary Fig. 2 it can be seen that the magnetic field in the center of the entering vortex is ~2*H*_0_, as expected for slowly moving (non-relativistic) Josephson vortex.

Three video files representing different dynamic regimes in the junction.

The Supplementary Movie 1 shows the most interesting case of the bifurcation point between 1/0 states at *H* = −0.55, which corresponds to the largest dip in Fig. 3i. Here an extra Josephson vortex enters and leaves the junction every period of tip oscillation. It occurs at the left side of the junction, where the tip is located. This is accompanied by significant flux-flow voltage generation, leading to dissipation and damping of tip oscillations.

The Supplementary Movie 2 represents simulations done for the same parameters at nearby field *H* = −1, which is away from the bifurcation point. Here the junction remains firmly in the 0-state, despite the same amplitude of the tip field. The total dissipation is non-zero, but significantly less than at the bifurcation point. The same happens at the other side from the bifurcation point in the 1-state and at all other *n*/*n* + 1 bifurcation points, as can be seen from Fig. 3i.

Supplementary Movie 3 shows an example of unidirectional ratchet-like vortex motion. Unlike all other presented simulations this one was done for a much larger tip amplitude (as seen from the bottom panel in the video) and lower damping *α* = 0.1. Ratchet-like behavior occurs also for previous parameters, but it is much less pronounced.

Influence of the tip-device distance. In Supplementary Fig. 3 MFM maps acquired at different distances (lifts) between the tip and the device are presented. At very short distances the magnetic field of the tip is high: It induces both Abrikosov and Josephson vortices. At higher lifts only Josephson vortices are generated. As the total magnetic flux created by the tip decreases with increasing the tip-device distance, the number of generated Josephson vortices lowers. This is confirmed by the increasing distance between rings/arcs. See Supplementary Movie 4.

Influence of the external magnetic field. Supplementary Fig. 4 displays MFM maps taken at different intensities of the external magnetic field. The external magnetic field induces an additional magnetic flux through the junction and modifies the number of generated Abrikosov and Josephson vortices. Thus, the tip and the external field produce similar effects; the total field being the sum of the two contributions, as discussed in the main text. This further confirms the results presented in Fig. 4 of the main manuscript. See Supplementary Movie 5.

Influence of temperature. In Supplementary Fig. 5 MFM phase maps acquired at different temperatures are shown. The main effect here is an “expansion” of rings/arcs when the temperature is increased. No Josephson vortices are observed above *T*_c_. The phenomenon is related to the temperature evolution of London penetration depth *λ*_L_ ~ 1/(1 − (*T*/*T*_c_)^4^)^1/2^. *λ*_*L*_ increases with temperature and modifies the distribution of screening currents and generated diamagnetic fields, thus relaxing both kinetic and magnetic energy. See Supplementary Movie 6.

### Dissipation and phase shift in MFM

In scanning probe microscopies, the fine tracking of parameters of the resonant cantilever-tip circuit often enables an enhanced sensitivity. Recently, the approach was successfully applied to reveal and modify the charge state of individual quantum dots through the electrostatic interaction^56^, reaching the detection limit of a single electron charge. In our case, the link between the calculated dissipation in Fig. 3 and the observed phase shifts (Figs. 2 and 4) is due to the magnetic interaction between the MFM tip and the device. As the oscillating screening currents and Josephson vortices are generated in the device, the tip experiences an additional oscillating force *F*_z_ = *F*_0_*cos*(*ωt*) at a frequency *ω* close to its resonant frequency *ω*_0_. In the case of small deflections, the tip-cantilever can be modeled by a damped harmonic oscillator with a proof mass *m*, and a spring constant *k*. It oscillates as *z* = *z*_0_*cos*(*ωt* + *θ*), *θ* being the phase shift between the force and the tip displacement. In the presence of a non-zero *z*−component of the force gradient, the oscillation amplitude *z*_0_ and the phase shift *θ* change by^57^:

3 $$\delta z \approx \left( {\frac{{2z_0Q}}{{3\sqrt 3 k}}} \right)\frac{{\partial F}}{{\partial z}},\delta \theta \approx \frac{Q}{k}\frac{{\partial F}}{{\partial z}},$$

where $Q = \frac{{kz_0^2\omega _0}}{{2P_{{\mathrm{dis}}}}}$ is the quality factor of the cantilever, and *P*_dis_ is the dissipated power. For a typical MFM cantilever used in this work, *k* = 2.8 N m^−1^, *z*_0_ = 20 nm, *ω*_0_*/*2*π* = 100 kHz, *Q* ~ 4000, it gives *P*_dis_ ~ 8.8 × 10^−14^ W. From Eq. (1) one obtains that the phase drops by *δθ* ~ 2 deg (a typical value in our experiments, Fig. 2) correspond to variations in the oscillation amplitude by $\delta z = 2z_0\delta \theta /(3\sqrt 3 )\sim 0.3$ nm (~1.5%). Consequently, the dissipated power *P*_dis_ changes by $\delta P_{{\mathrm{dis}}} = P_{{\mathrm{dis}}}2\delta z/z_0 = P_{{\mathrm{dis}}}4\delta \theta /(3\sqrt 3 )\sim 2.6 \cdot 10^{ - 15}\,{\mathrm{W}}$, with a linear link between the variations of the dissipation and the phase shifts. This number is consistent with the experimentally obtained result presented in Supplementary Fig. 6.

In Supplementary Fig. 6a, b the frequency map and the excitation voltage map were measured with the phase-locked loop (PLL) using the amplitude control mode of the MFM. In this mode the cantilever oscillation amplitude *z*_0_ is kept constant by adjusting the cantilever excitation voltage *A*_exc_; the latter is connected to the amplitude of the cantilever oscillations as *A*_exc_ = *z*_0_/*Q*. From the variations *δA*_exc_ it is possible to estimate the modification of the *Q*-factor of the system *δQ* = −*QδA*_exc_/*A*_exc_ and then evaluate the variation of dissipation power *δP*_dis_, as follows. The full dissipation power in the cantilever is $P_{{\mathrm{dis}}} = \frac{{kz_0^2\omega _0}}{{2Q}}$ (see the section Methods of the main manuscript). Consequently, the variation of the dissipation power is $\delta P_{{\mathrm{dis}}} = - \delta Q\frac{{kz_0^2\omega _0}}{{2Q^2}} = - P_{{\mathrm{dis}}}\delta Q/Q = P_{{\mathrm{dis}}}\delta A_{{\mathrm{exc}}}/A_{{\mathrm{exc}}}$. Typical parameters of our cantilevers are *k* = 2.8 N m^−1^, *z*_0_ = 20 nm, *ω*_0_/2*π* = 100 kHz and *Q* = 4000 (estimated from the resonance curve (Supplementary Fig. 6c). It gives *P*_dis_ ~ 8.8 × 10^−14^ W. In Supplementary Fig. 6 at the bifurcation points typical numbers are *A*_exc _= 12 mV, *δA*_exc_ = 0.3 mV. Therefore the variation of the dissipation power of the cantilever due to Josepshon vortex flux-flow is *δP*_dis_ ~ 2.2 × 10^−15^ W. Note that this value is very close to *δP*_dis_ ~ 2.6 × 10^−15^ W estimated from the phase shifts in the section Methods of the main manuscript.

It is also in a good quantitative agreement. Quantitatively, the unit of flux-flow dissipation in numerical simulations presented in Fig. 3 of the main text corresponds to $10^{ - 3}I_{\mathrm{c}}^2R_{\mathrm{n}} \simeq 51\,{\mathrm{pW}}$, and the maximum flux-flow power can rich 20 times this value, see Fig. 3f, i.e. typically from 0.1 up to 1 nW. However, to speed-up simulations they were made for the tip angular frequency *ω* = 0.05*ω*_p_ which is much larger than the actual tip frequency in MFM experiment. In order to obtain a relevant number for comparison with experiment we need to downscale the calculated dissipation power to the relevant experimental frequency. As described above and can be seen from the Supplementary Video 1, the dissipation peaks correspond to entrance and exit of one Josephson vortex every cycle of tip oscillations. The entrance/exit times are determined by characteristics times of the junction, and are not related to the tip frequency (provided it is much smaller than all characteristic frequencies in the junction). In this case the total energy dissipated per cycle is approximately constant. Therefore, when the tip frequency is reduced, the dissipated power will reduce proportionally to the tip frequency. We have checked this numerically for a few selected points. Thus, predicted dissipation for the experimental tip frequency *f*_exp_ should be scaled as *P*_exp_ = *P*_*sim*_*f*_exp_/*f*_sim_. To make this estimation we need to calculate the plasma frequency $\omega _{\mathrm{p}} = \sqrt {\frac{{2\pi I_{\mathrm{c}}}}{{{\mathrm{\Phi }}_0C}}}$, where *C* is the junction capacitance. Unfortunately, an accurate estimation of the stray capacitance for our planar junction is rather difficult. Generally it is small, of order *C* ~ 1 pF. Taking *f*_exp_ ≃ 100 kHz, *f*_sim_ = 0.05*ω*_p_/2*π* and *C* = 1 pF we obtain *f*_exp_/*f*_sim_ ≃ 3.5 × 10^−6^ and predicted experimental values for the vortex-induced tip dissipation *P*_FF_ ~ 0.4−4 fW, consistent with the excess tip dissipation *δP*_dis_ estimated above.

The above calculations also show that a MFM could be used as a very sensitive local wattmeter.

## Supplementary information

Supplementary Information

Description of Additional Supplementary Files

Supplementary Movie 1

Supplementary Movie 2

Supplementary Movie 3

Supplementary Movie 4

Supplementary Movie 5

Supplementary Movie 6

## Data Availability

Authors can confirm that all relevant data are included in the paper and its supplementary information files. Additional data are available on request from the authors.
