# Author Correction: Local Josephson vortex generation and manipulation with a Magnetic Force Microscope

**DOI:** 10.1038/s41467-019-12356-6

**Published:** 2019-10-07

**Authors:** Viacheslav V. Dremov, Sergey Yu. Grebenchuk, Andrey G. Shishkin, Denis S. Baranov, Razmik A. Hovhannisyan, Olga V. Skryabina, Nickolay Lebedev, Igor A. Golovchanskiy, Vladimir I. Chichkov, Christophe Brun, Tristan Cren, Vladimir M. Krasnov, Alexander A. Golubov, Dimitri Roditchev, Vasily S. Stolyarov

**Affiliations:** 1 Moscow Institute of Physics and Technology, 141700 Dolgoprudny, Russia; 2 Dukhov Research Institute of Automatics (VNIIA), 127055 Moscow, Russia; 3 Institute of Solid State Physics RAS, 142432 Chernogolovka, Russia; 4 LPEM, ESPCI Paris, PSL Research University, CNRS, 75005 Paris, France; 5 National University of Science and Technology MISIS, 119049 Moscow, Russia; 6 Institut des Nanosciences de Paris, INSP, UMR-7588, Sorbonne University, CNRS, 75005 Paris, France; 7 Department of Physics, Stockholm University, AlbaNova University Center, SE-10691 Stockholm, Sweden; 8 Faculty of Science and Technology and MESA+ Institute of Nanotechnology, 7500AE Enschede, The Netherlands; 9 Sorbonne Universite, CNRS, LPEM, 75005 Paris, France; 10 Donostia International Physics Center (DIPC), 20018 San Sebastin/Donostia, Basque, Spain; 11 Solid State Physics Department, Kazan Federal University, 420008 Kazan, Russia

**Keywords:** Superconducting properties and materials, Superconducting devices, Atomic force microscopy

Correction to: *Nature Communications* 10.1038/s41467-019-11924-0, published online 05 September 2019.

The original version of this Article contained an error in the spelling of the author Christophe Brun, which was incorrectly given as Chritophe Brun.

Additionally this Article omitted from the author list the seventh author Nickolay Lebedev, who is from the ‘Moscow Institute of Physics and Technology, 141700 Dolgoprudny, Russia’. These errors have now been corrected in both the PDF and HTML versions of the Article.

