# Supplementary material for: Local Josephson vortex generation and manipulation with a Magnetic Force Microscope
Source: Nat Commun. 2019 Sep 5;10:4009. doi: 10.1038/s41467-019-11924-0 (PMC6728352; doi:10.1038/s41467-019-11924-0)
Supplement: Supplementary file 1 — Supplementary Information [file 41467_2019_11924_MOESM1_ESM.pdf]

Supplementary Information.
Local Josephson vortex generation and detection with a Magnetic Force Microscope

Viacheslav V. Dremov, Sergey Yu. Grebenchuk, Andrey G. Shishkin, Denis S. Baranov, Razmik A. Hovhannisyan, Olga V. Skryabina, Nikolay M. Lebedev, Igor A. Golovchansky, Vladimir I. Chichkov, Chritophe Brun, Tristan Cren, Vladimir M. Krasnov, Alexander A. Golubov, Dimitri Roditchev & Vasily S. Stolyarov

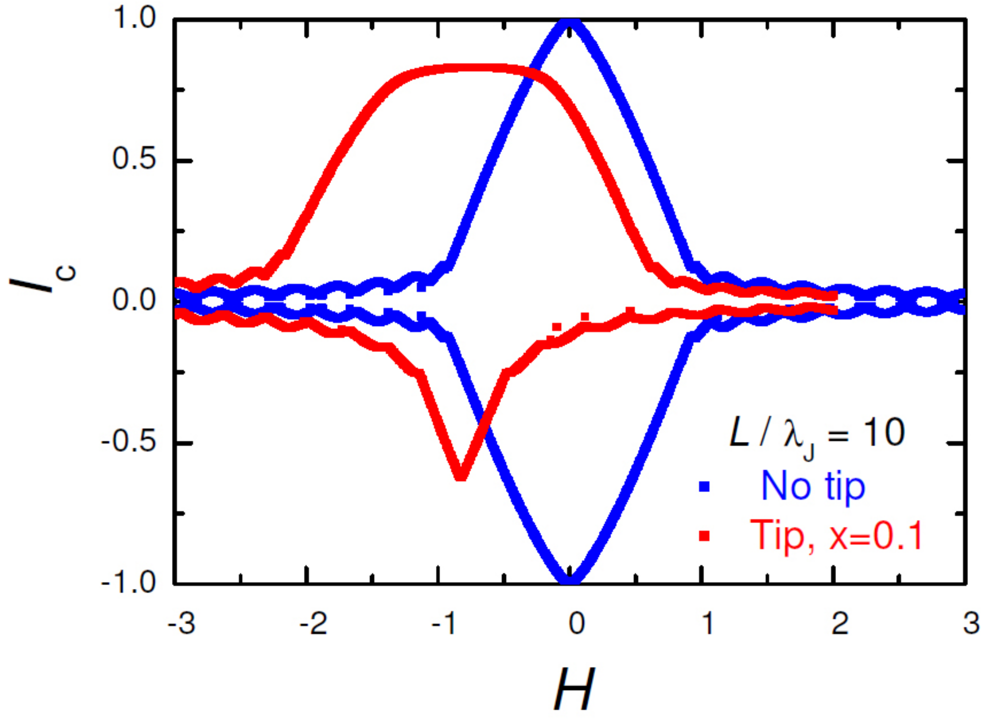

Supplementary Figure 1: Simulated $I_c(H)$ patterns for a long junction $L/\lambda_J = 10$ without MFM tip (blue symbols) and with a static tip at the left edge of the junction $x = 0.1\ \lambda_J$, $y = z = 0$ (red symbols). It is seen that the tip field shifts and distorts the $I_c(H)$ pattern.

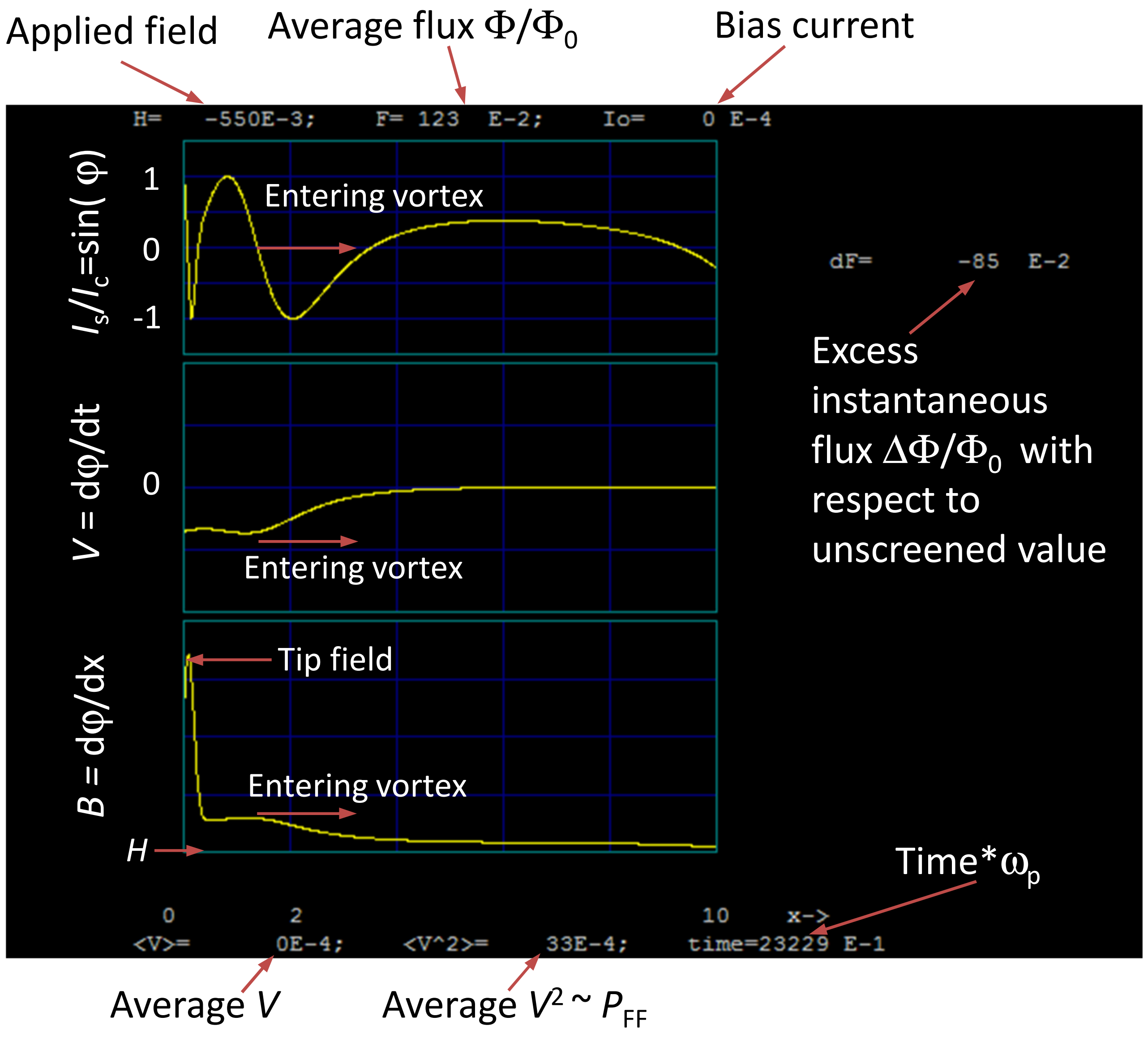

Supplementary Figure 2: Information about Supplementary Movie files 1-3 with numerical simulations of vortex dynamics. Top panel: spatial distribution of supercurrent density sin(φ), middle panel: generated voltage, bottom panel: magnetic field. The frame is taken from the Supplementary Movie 1 and clearly indicates penetration of a Josephson vortex from the left side of the junction, where the tip is located.

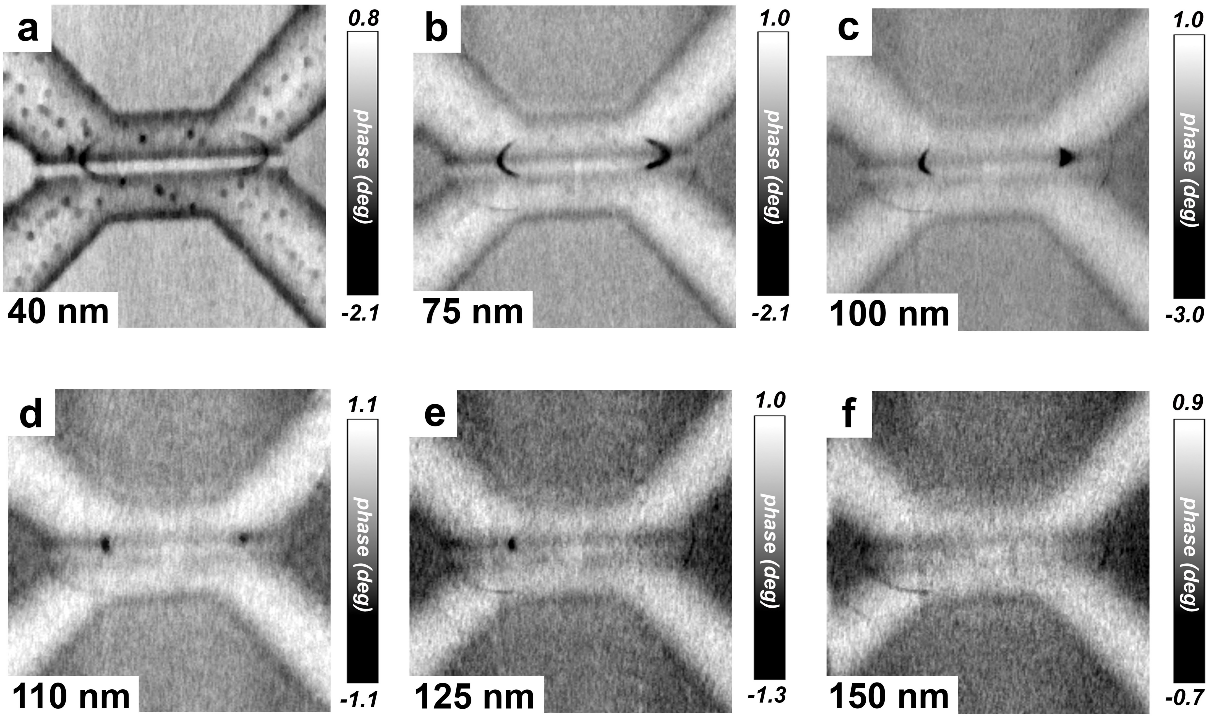

Supplementary Figure 3: MFM maps acquired at different distances (lifts) between the tip and the device ($T = 4.3$ K, $H_{\mathrm{ext}} = 0$, $I_{\mathrm{ext}} = 0$). The numbers indicate the lifts starting from Nb surface.

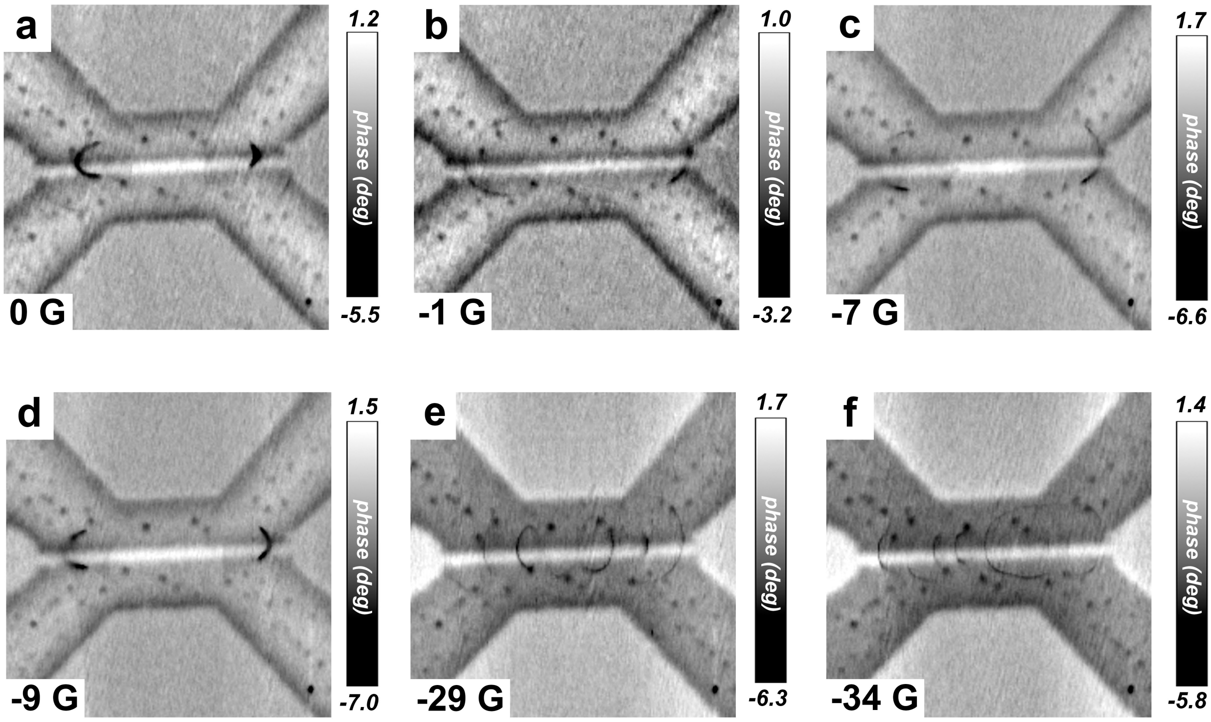

Supplementary Figure 4: MFM maps acquired at different values of an externally applied magnetic field ($T = 4.3$ K, $I_{ext} = 0$, fixed lift). The external magnetic field adds to the field of the tip and induces more Josephson vortices in the device.

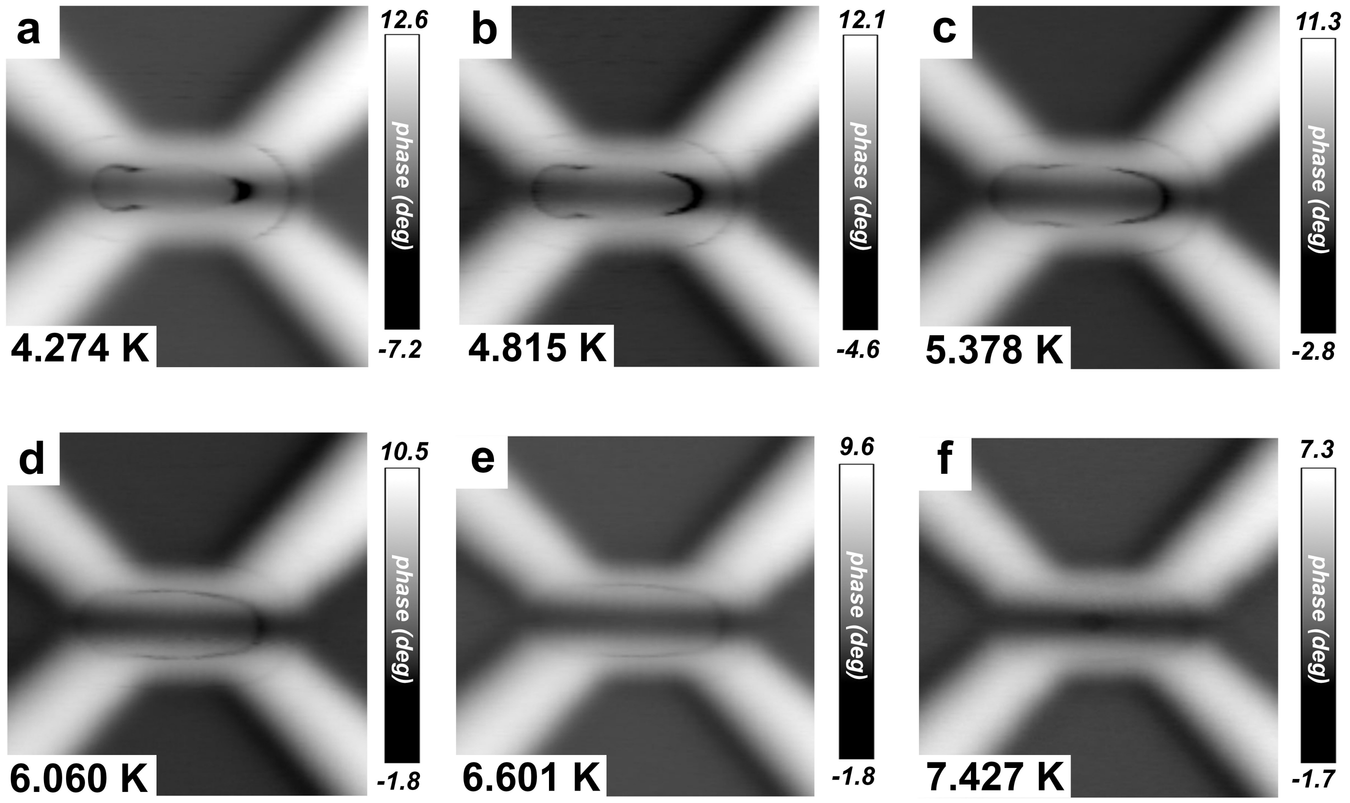

Supplementary Figure 5: MFM maps acquired at different temperatures ($H_{ext} = 0, I_{ext} = 0$, fixed lift). The main effect is an expansion of rings/arcs as the temperature is increased.

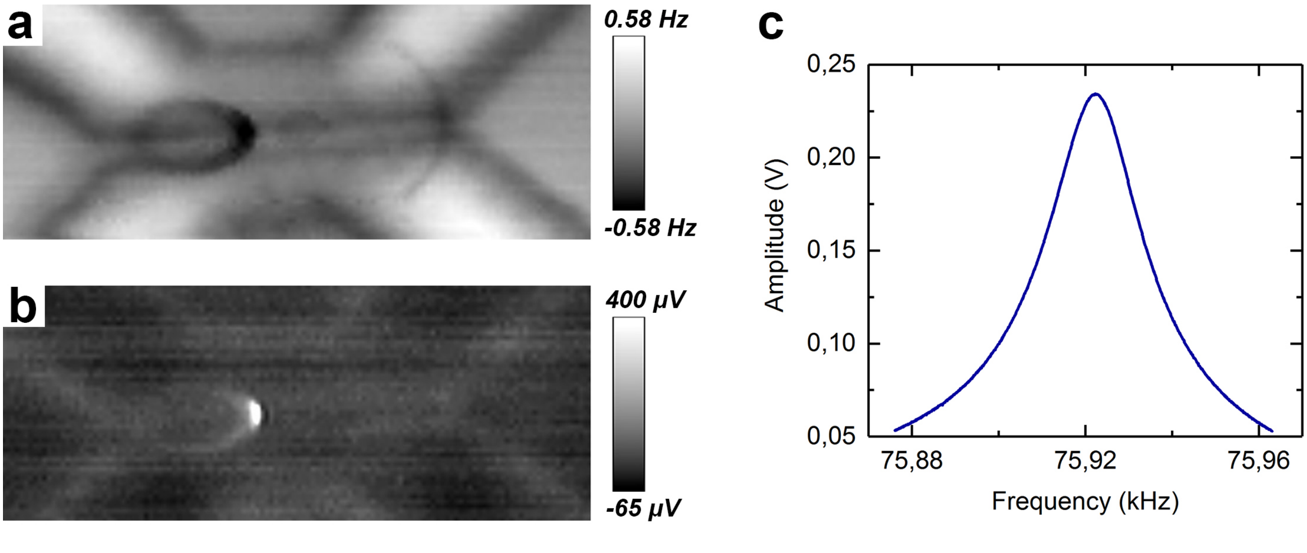

Supplementary Figure 6: MFM-maps of frequency shift **a** and excitation voltage shift **b**. These images were measured with phase locked loop (PLL) with constant amplitude. **c** - resonance curve of the cantilever's oscillations.
