# Supplementary material for: Local Josephson vortex generation and manipulation with a Magnetic Force Microscope
Source: Nat Commun. 2019 Sep 5;10:4009. doi: 10.1038/s41467-019-11924-0 (PMC6728352; doi:10.1038/s41467-019-11924-0)
Supplement: Supplementary file 2 — Description of Additional Supplementary Files [file 41467_2019_11924_MOESM2_ESM.pdf]

Description of Additional Supplementary Files

File Name: Supplementary Movie 1
Description: The Supplementary Movie 1 shows the most interesting case of the bifurcation point between 1/0 states at H=-0.55, which corresponds to the largest dip in Fig.3i. Here an extra Josephson vortex enters and leaves the junction every period of tip oscillation. It occurs at the left side of the junction, where the tip is located. This is accompanied by significant flux-flow voltage generation, leading to dissipation and damping of tip oscillations.

File Name: Supplementary Movie 2
Description: The Supplementary Movie 2 represents simulations done for the same parameters at nearby field H=-1, which is away from the bifurcation point. Here the junction remains firmly in the 0-state, despite the same amplitude of the tip field. The total dissipation is non-zero, but significantly less than at the bifurcation point. The same happens at the other side from the bifurcation point in the 1-state and at all other n/n+1 bifurcation points, as can be seen from Fig.3i.

File Name: Supplementary Movie 3
Description: Supplementary Movie 3 shows an example of unidirectional ratchet-like vortex motion. Unlike all other presented simulations this one was done for a much larger tip amplitude (as seen from the bottom panel in the video) and lower damping $\alpha=0.1$. Ratchet-like behavior occurs also for previous parameters, but it is much less pronounced.

File Name: Supplementary Movie 4
Description: Influence of the tip-device distance. In Supplementary Figure 3 MFM maps acquired at different distances (lifts) between the tip and the device are presented. At very short distances the magnetic field of the tip is high: It induces both Abrikosov and Josephson vortices. At higher lifts only Josephson vortices are generated. As the total magnetic flux created by the tip decreases with increasing the tip-device distance, the number of generated Josephson vortices lowers. This is confirmed by the increasing distance between rings/arcs.

File Name: Supplementary Movie 5
Description: Influence of the external magnetic field. Supplementary Figure 4 displays MFM maps taken at different intensities of the external magnetic field. The external magnetic field induces an additional magnetic flux through the junction and modifies the number of generated Abrikosov and Josephson vortices. Thus, the tip and the external field produce similar effects; the total field being the sum of the two contributions, as discussed in the main text. This further confirms the results presented in Fig.4 of the main manuscript.

File Name: Supplementary Movie 6
Description: Influence of temperature. In Supplementary Figure 5 MFM phase maps acquired at different temperatures are shown. The main effect here is an "expansion" of rings/arcs when the temperature is increased. No Josephson vortices are observed above T_c. The phenomenon is related to the temperature evolution of London penetration depth $\lambda_L \sim 1/(1-(T/T_c)^4)^{0.5}$. λ_L increases with temperature and modifies the distribution of screening currents and generated diamagnetic fields, thus relaxing both kinetic and magnetic energy. See Supplementary Movie~6.
