# Supplementary figures and images for: Local Josephson vortex generation and manipulation with a Magnetic Force Microscope
Source: Nat Commun. 2019 Sep 5;10:4009. doi: 10.1038/s41467-019-11924-0 (PMC6728352; doi:10.1038/s41467-019-11924-0)

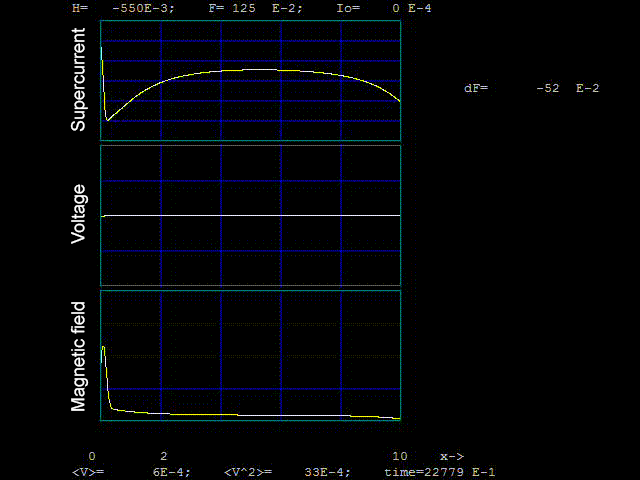

Supplement: Supplementary file 3 — Supplementary Movie 1 [file 41467_2019_11924_MOESM3_ESM.gif]

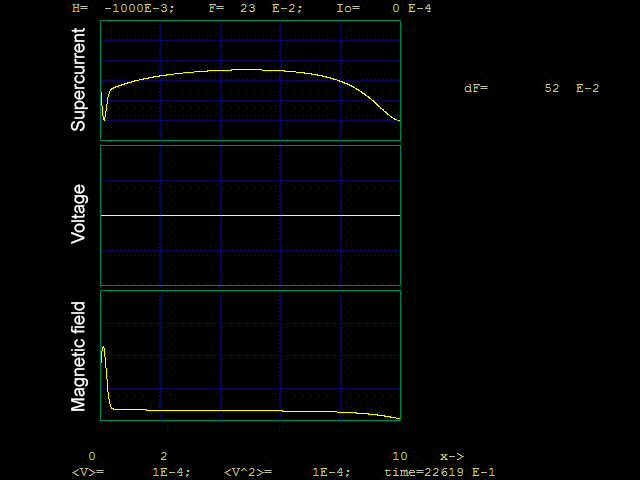

Supplement: Supplementary file 4 — Supplementary Movie 2 [file 41467_2019_11924_MOESM4_ESM.gif]

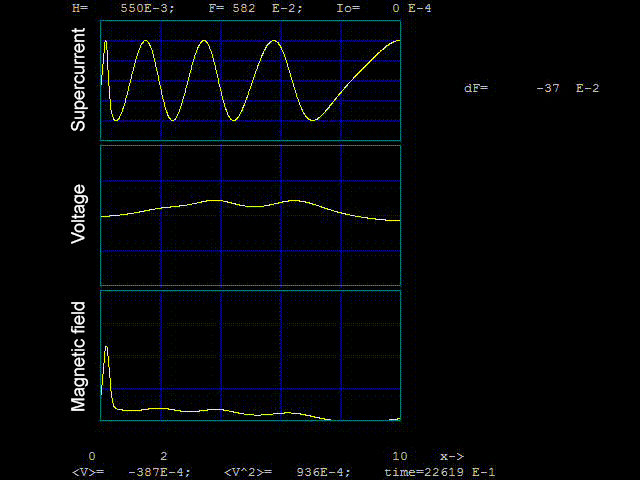

Supplement: Supplementary file 5 — Supplementary Movie 3 [file 41467_2019_11924_MOESM5_ESM.gif]

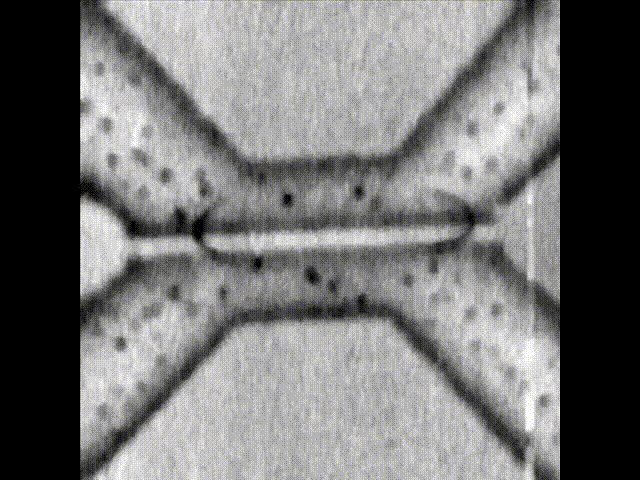

Supplement: Supplementary file 6 — Supplementary Movie 4 [file 41467_2019_11924_MOESM6_ESM.gif]
